# Surface morphology and *in vitro* leachability of soft liners modified by the incorporation of antifungals for denture stomatitis treatment

**DOI:** 10.1590/1678-7757-2020-0639

**Published:** 2020-02-26

**Authors:** Adelaida SÁNCHEZ-ALIAGA, Paulo Vitor FARAGO, Milton Domingos MICHÉL, Carolina Yoshi Campos SUGIO, Karin Hermana NEPPELENBROEK, Vanessa Migliorini URBAN

**Affiliations:** 1 Universidade Estadual de Ponta Grossa Departamento de Odontologia Ponta GrossaParaná Brasil Universidade Estadual de Ponta Grossa, Departamento de Odontologia, Ponta Grossa, Paraná, Brasil.; 2 Universidade Estadual de Ponta Grossa Departamento de Ciências Farmacêuticas Ponta GrossaParaná Brasil Universidade Estadual de Ponta Grossa, Departamento de Ciências Farmacêuticas, Ponta Grossa, Paraná, Brasil.; 3 Universidade Estadual de Ponta Grossa Departamento de Engenharia de Materiais Ponta GrossaParaná Brasil Universidade Estadual de Ponta Grossa, Departamento de Engenharia de Materiais, Ponta Grossa, Paraná, Brasil.; 4 Universidade de São Paulo Faculdade de Odontologia de Bauru Departamento de Prótese e Periodontia BauruSão Paulo Brasil Universidade de São Paulo, Faculdade de Odontologia de Bauru, Departamento de Prótese e Periodontia, Bauru, São Paulo, Brasil.

**Keywords:** Anti-infective agents, Drug release, Denture liners, Stomatitis, denture, Surface properties

## Abstract

**Objective:**

To evaluate the surface morphology and in vitro leachability of temporary soft linings modified by the incorporation of antifungals in minimum inhibitory concentrations (MIC) for *Candida albicans* biofilm.

**Methodology:**

Specimens of soft lining materials Softone and Trusoft were made without (control) or with the addition of nystatin (Ny), miconazole (Mc), ketoconazole (Ke), chlorhexidine diacetate (Chx), or itraconazole (It) at their MIC for *C. albicans* biofilm. The surface analyses were performed using Confocal laser scanning microscopy after 24 h, 7 days, or 14 days of immersion in distilled water at 37ºC. *In vitro* leachability of Chx or Ny from the modified materials was also measured using Ultraviolet visible spectroscopy for up to 14 days of immersion in distilled water at 37ºC. Data (μg/mL) were submitted to ANOVA 1-factor/Bonferroni (α=0.05).

**Results:**

Softone had a more irregular surface than Trusoft. Morphological changes were noted in both materials with increasing immersion time, particularly, in those containing drugs. Groups containing Chx and It presented extremely porous and irregular surfaces. Both materials had biexponential release kinetics. Softone leached a higher concentration of the antifungals than Trusoft (p=0.004), and chlorhexidine was released at a higher concentration than nystatin (p<0.001).

**Conclusions:**

The surface of the soft lining materials changed more significantly with the addition of Chx or It. Softone released a higher concentration of drugs than Trusoft did, guiding the future treatment of denture stomatitis.

## Introduction

Denture-induced stomatitis is considered the most common fungal infection among denture wearers.^1^This pathology is primarily associated with infection by *Candida albicans* , which is found in 50 to 98% of all cases.^2 - 3^ Treatments for denture stomatitis are varied and include topical and systemic antifungal therapy, oral hygiene care, procedures for denture cleaning and disinfection, replacement of old dentures, removal of anatomical irregularities, reestablishment of nontraumatic occlusion, and nutritional restitution.^1 - 5^ Additionally, to protect and preserve the mucosal integrity, the patients should sleep without the dentures.^1 , 5 , 6^ Studies *in vivo* have reported that the nocturnal wear increases the colony counts of *C. albicans* , which reinforces that such habit can induce denture stomatitis.^6^

Topical antifungal agents are widely used in the therapy for this condition.^1^ However, their effectiveness can be compromised by many factors, including lack of patient perception of the infection, costs required for the medication, continuous denture wear, unpleasant taste, and patient compliance in strictly following the posology.^2^ Furthermore, salivary flow, tongue movements, and swallowing decrease drug concentration to subtherapeutic doses.^2^ However, systemic administration of antifungal agents should be carefully administered these drugs can induce hepatotoxic and nephrotoxic effects.^4^

*Candida* spp. colonization is predominantly more observed on the internal surfaces of removable dentures than on denture-bearing epithelium^2 , 3 , 5^ due to the high affinity between microorganisms and acrylic resin.^7^ It has been demonstrated that the average depth of *C. albicans* in denture base resins varies according to the time of exposure to fungal contamination, reaching 631 μm at 21 days.^7^ Therefore, the treatment of denture stomatitis should focus on dentures, which may act as the primary source of mucosal reinfection.^3 , 5^ Since denture base acrylic resin is likely to be penetrated by *Candida* , especially in recurrent denture-induced stomatitis, it was suggested the removal of the at least a 1-mm layer of contaminated resin from the infected denture-fitting surfaces.^7^In this regard, incorporation of antifungal agents into denture base materials for gradual release to the oral cavity^8^ can prevent biofilm accumulation,^8^ inhibit *C. albicans* colonization,^8 , 9^ and thus, contribute to the treatment of denture-induced stomatitis.

In temporary soft lining materials, this modification has some advantages: reduction of trauma caused by the rigid internal surface of heat-cured acrylic resin of removable dentures; elimination of contact of the contaminated surface with oral tissues that leads to the reinfection cycle; and action of antifungal drugs incorporated in the material directly on infected tissues.^8 - 9^ In this context, denture stomatitis might be treated for two weeks, a period similar to the treatment with conventional topical antifungals and maximum period tolerated by these temporary soft materials due to their degradation and gradual stiffening. Since this treatment option does not depend on patient compliance,^8 - 9^ it may be especially beneficial for older patients with physical or mental disorders, or in institutional settings, where patients and staff cannot follow all recommended instructions to achieve a successful treatment.^2^

Bueno, et al.^10^ (2015) determined, by spectrophotometric analysis using tetrazolium salt reduction assay (XTT), the concentrations able to inhibit 90% or more of *C. albicans* growth (minimal inhibitory concentrations – MICs) for up to 14 days for five drugs when incorporated into two temporary soft denture liners (Softone and Trusoft). However, before using this protocol as a therapeutic option in individuals with denture stomatitis, it is necessary to obtain a polymeric matrix modified by the addition of antifungals that simultaneously does not present altered physical^11 , 12^ and mechanical^13 - 15^ properties and is effective in drug release.

The size, molecular weight, dispersion, and concentration of drug particles in the polymeric matrix and the drug properties of diffusion and solubilization in the medium over time are important factors influencing drug release.^16^ In addition to the important characteristics of the polymeric matrix used as vehicle for release, factors as polymer micromorphology, permeability, porosity, and drug-matrix interaction also influence the release patterns.^16^ Therefore, this study evaluated the surface morphology and *in vitro* leachability of temporary soft linings Softone^TM^ (S) and Trusoft^TM^ (T) modified by the incorporation of antifungals in their MICs for *C. albicans* biofilm. The following hypotheses were tested: 1. The incorporation of antifungals in the temporary soft linings would cause changes in their surface morphology, yielding different roughness values; 2. The materials would release different concentrations of antifungals; and 3. The drugs would present different mechanisms of release from both polymeric matrices.

## Methodology

### Materials

**Figure 1:**
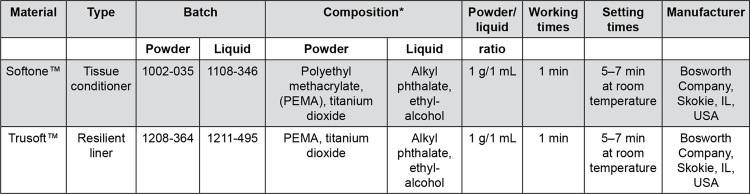
Soft lining materials chosen for this study

**Figure 2:**
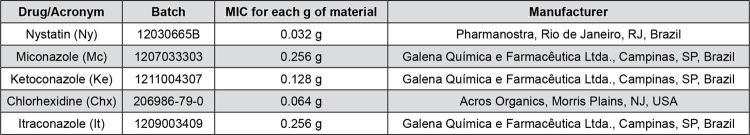
Antifungal agents and the previously determined minimum inhibitory concentrations for the biofilm of *C. albicans*

The temporary soft linings evaluated in this study are presented in Figure 1 . The antifungal drugs and their MICs against *C. albicans* biofilm determined in a previous study^10^ are shown in Figure 2 . MICs are presented as antifungal powder to each gram of soft lining material.

*According to the MSDS provided by the manufacturer.

### Specimen preparation

The amounts of antifungal powder ( Figure 2 ) were manually mixed to the powder of each soft lining until a homogeneous mixture was achieved.^16^ The liquids of the materials were then added and the material was mixed following the manufacturers’ instructions ( Figure 1 ). The material was then inserted in stainless steel molds and kept in place during the setting time recommended by the manufacturer ( Figure 1 ).

### Confocal laser scanning microscopy (CLSM)

Specimens (n=3; 10×5×4 mm) of the soft materials modified or not (control) by the addition of antifungals and of the control were fabricated and individually stored in distilled water at 37ºC during 24 h, 7 days, or 14 days. They were then dry-stored in an oven at 37ºC for additional 48 h.

The specimens were initially observed at 5× magnification to select the most representative area. Next, a total area of 1280 µm^2^ of each specimen was observed at 10× magnification, using green laser at a spectral range of 405 nm. The surface roughness, recorded as the Ra (mean roughness) parameter, was analyzed along three randomly drawn lines (cut off lambda C 80 µm). Small peaks and noises were removed by a Gaussian filter, and data were processed using software OLS 4000-BSW; Olympus. Data (RA) were submitted to ANOVA 3-factors/Bonferroni (α=0.05).

### *In vitro* leachability

To prepare the stock solutions (1,000 µg/mL), Chx was solubilized in methanol and Ny was dissolved in dimethyl formamide/methanol (1:10 v/v). The analytic solutions (0 to 25 µg/mL or 0 to 20 µg/mL) were then prepared to achieve the calibration curves of Chx and Ny, respectively. A mixture of reagents was added to the analytic solutions changing its color to orange (Chx) according to the method proposed by Holbrook^17^(1958) or to pink (Ny) in agreement with the method by Amer and Habeeb^18^(1975) with saturation proportional to the drug concentration in the sample. Before the analyses, the solutions passed through a filter with 0.45-µm pore size. Readings were performed in sextuplicate in increasing order of concentration using a UV-Vis spectrophotometer (Genesys 10 UV scanning; Thermo scientific) at 480 nm and 520 nm for Chx and Ny analyses, respectively.

Specimens (n=6; 50-mm diameter × 2-mm thickness) of soft materials containing Chx and Ny or not (control) were fabricated and individually stored in flasks with 50 mL of distilled water at 37ºC for up to 14 days. For Chx, the volume of distilled water used for immersion of specimens was sufficient to maintain the sink conditions of the medium. Since Ny is nearly insoluble in water, it was necessary to add 2% (1 g) of surfactant sodium lauryl sulfate. This surfactant caused a little impact on drug release.

Daily, aliquots of 1 mL of each storage solution of specimens were collected to analyze the drug release from the soft materials. This aliquot received addition of the same reagents, following the protocols used for preparing analytical solutions. After each day, the same volume of the aliquot removed for analysis was refilled with an equal volume of distilled water without or with 2% of sodium lauryl sulfate in the case of Ny. Quantification of drug concentration released from the materials was performed by using the linear regression equations obtained from the calibration curves. The percentage of daily release was estimated compared to the initial quantity of drug added to the specimens, and the release profile was obtained compared to the total period of 14 days. Data (μg/mL) were submitted to ANOVA 1-factor/Bonferroni (α=0.05).

The release kinetics was also mathematically analyzed by adjusting the experimental data to the monoexponential model (Equation 1), to the biexponential model (Equation 2), to the zero order model (Equation 3), and to the Weibull model (Equation 4), considering the results of the model selection criteria (MSC), correlation coefficient, graphic adjustment, and coherence of values found for the velocity constants of each model, using the software GraphPad Prism 5 (GraphPad Software Inc.).

$$\%D=100\cdot \left(1-e^{-kt}\right)$$ (1)

$$\%D=100\cdot \left[1-\left(Ae^{-at}+Be^{-\beta t}\right)\right]$$ (2)

D=k⋅t (3)

$$D=100\cdot \left\{1-e^{-[(t/td)bJ}\right\}$$ (4)

in which: “% *D”* is the percentage of drug dissolved over time *“t”* ; “ *k”* , “α”, and “β” are dissolution kinetic constants observed; “ *A”* and “ *B”* are the initial drug concentrations that contribute to the two dissolution stages; “ *td”* is the time at which 63.2% of the drug are dissolved; “ *b”* is the parameter related to the structural and geometric characteristics of the pharmaceutical product.

Based on the release data achieved, the mechanisms involved in the process of drug release from each polymeric matrix were also analyzed, using the software Scientist^®^ 3.0 *for Windows* (Micromath^®^). The semi-empirical Korsmeyer-Peppas model^19^ was used to extend the information about the mechanism of drug release from the polymeric matrix. This method is based on the power law, which exponentially relates the drug release with time, and should be applied to the first 60% of drug released (Equation 5):

$$ft=a,t^{n}$$ (1)

in which: “ *a* ” is the constant that incorporates the structural and geometric characteristics of the pharmaceutical product; “n” is the exponent of release that indicates the mechanism of release; “ƒt” is the drug fraction dissolved at time “ *t* ”.

### Scanning electron microscopy (SEM)

SEM images of antifungal drugs were obtained to complement the other analyses. Small random amounts of powder of each antifungal were fixated on metallic stubs and sputter-coated with gold-palladium using an ion coater (IC-50 Ion Coater; Shimadzu) in a vacuum environment. Each sample was assessed by SEM (SSX 550 Superscan; Shimadzu) at an acceleration voltage of 20 KV, scanning of 100 s, and surface area of 25 µm^2^.^16^ The most representative images were selected to illustrate the characteristics of each antifungal.

## Results

### Confocal laser scanning microscopy (CLSM)

**Figure 3:**
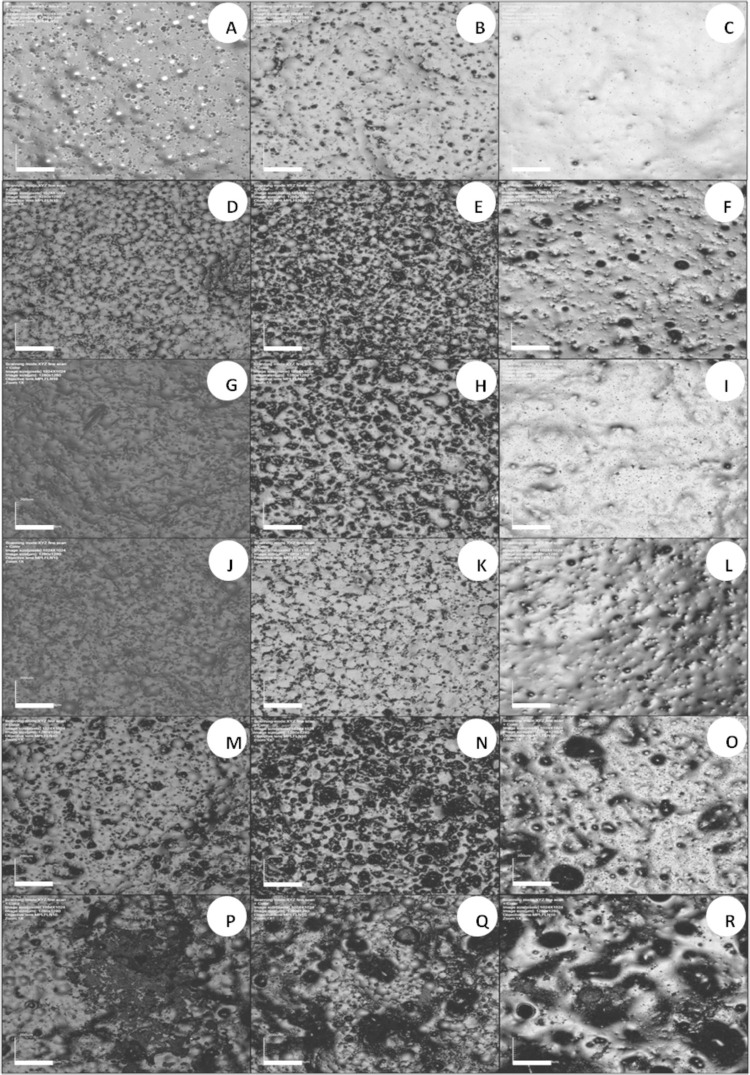
CLSM images of tissue conditioner Softone at 24-h (1st column), 7-day (2nd column), and 14-day intervals (3rd column). A to C – control; D to F – Ny; G to I – Mc; J to L – Ke; M to O – Chx; and P to R – It. Scale bar = 200 µm (Magnification 10x)

**Figure 4:**
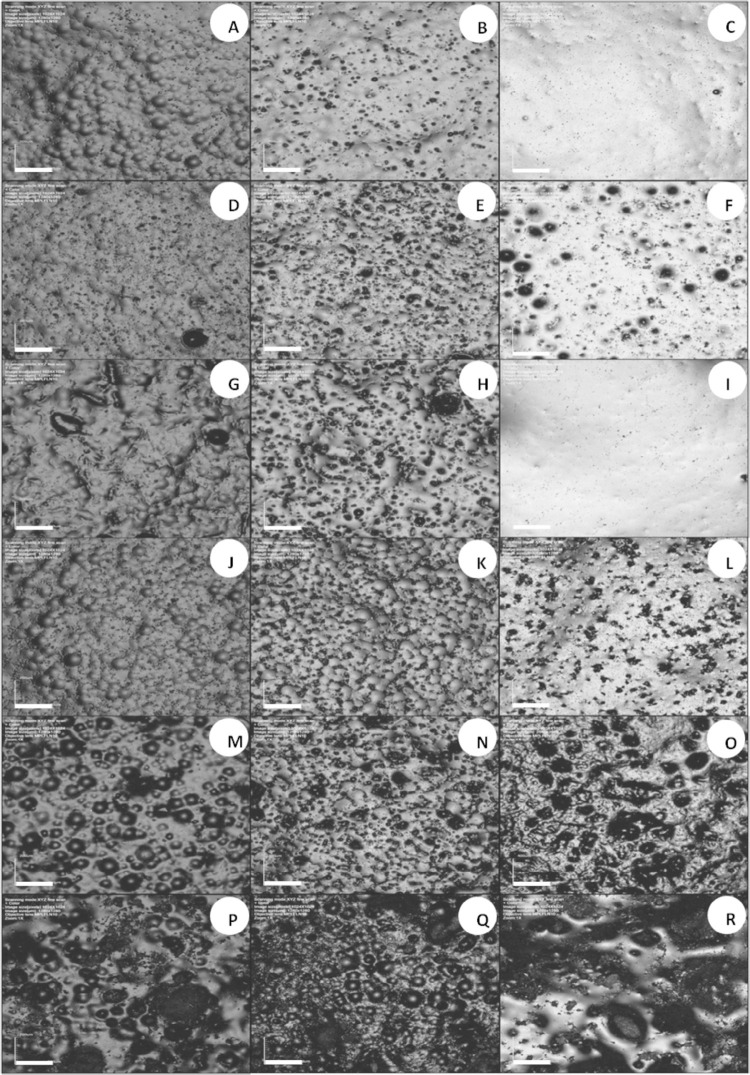
CLSM images of resilient liner Trusoft at 24-h (1st column), 7-day (2nd column), and 14-day intervals (3rd column). A to C – control; D to F – Ny; G to I – Mc; J to L – Ke; M to O – Chx; and P to R – It. Scale bar = 200 µm (Magnification 10x)

Figures 3 and 4 present CLSM images of specimens of all groups in immersion times of 24 h, 7 days, and 14 days for Softone and Trusoft, respectively. The control groups presented similar surface characteristics. At 24 h, they presented irregular surfaces exhibiting pearls and small pores distributed on the surface ( Figures 3A and 4A ). At seven days, there was a reduction in the quantity of pearls, which were also more subtle; conversely, there was an increase in the quantity and size of pores on the surface ( Figures 3B and 4B ). At 14 days, the surfaces were smoother, yet they presented a swollen aspect ( Figures 3C and 3C ).

Only groups containing Mc ( Figures 3G to 3I and 4G to 4I ) presented closer features to the control groups, yet they presented more irregular surfaces at seven days. The specimens modified by the addition of Ny still presented pearls and increased quantity and size of pores at seven days (Figures 3E and 4E). At 14 days, the diameters of pores were increased ( Figures 3F and 4F ). For groups with incorporation of Ke, a flat and porous surface was observed for Softone specimens at 7-day period ( Figure 3K ). The Trusoft specimens had fewer pearls and more pores at seven days ( Figure 4K ). At 14 days, Softone modified by Ke exhibited a swollen surface with larger pores ( Figure 3L ). A flatter surface, containing small pores, was observed for Trusoft specimens modified by Ke ( Figure 4L ).

The groups containing Chx and It exhibited the greatest changes in surface morphology (Figures 3M to 3R and 4M to 4R). Softone modified by Chx had more quantity of pores on the surface ( Figure 3M ). There was an increase in the amount and size of pores at day seven for both materials ( Figures 3N and 4N ) that was greater for Softone ( Figure 3N ). At 14-day period, the surface of Softone was smoother with a swollen aspect, yet porous ( Figure 3O ), and the surface of Trusoft was irregular with greater quantity of pores ( Figure 4O ). The specimens with addition of It exhibited completely irregular surfaces, containing pearls, pores, and spicules at days one (Figures 3P and 4P) and seven, that were even more irregular at 7-day period ( Figures 3Q and 4Q ). At day 14, the surface was swollen and irregular, exhibiting spicules and large pores ( Figures 3R and 4R ).

**Figure 5:**
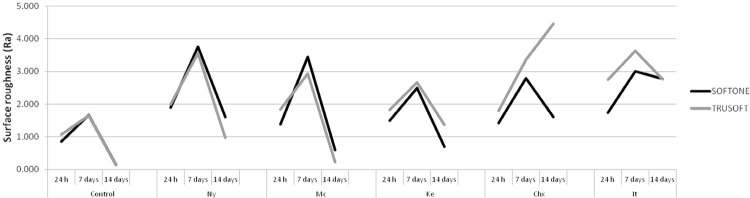
Mean roughness values (Ra) obtained for Softone and Trusoft in the evaluated groups

The mean roughness (Ra) results are presented in Figure 5 . Higher roughness values were observed in most groups modified by the incorporation of antifungals compared with those from the control groups at either 7- or 14-day periods (p<0.003). The roughness values of Softone and Trusoft specimens modified by the addition of Mc as well as of Softone specimens modified by Ke were not different from those of the control groups (p>0.186) at day 14. Except for Softone control group (p=1.000), Ra values of all other specimens increased at day seven (p<0.026). At the 14-day interval, the roughness decreased to values as low as those observed at 24-hour interval on specimens from control, Ny, Mc, and Ke groups (p<0.001). Similar outcome was observed on either Softone specimens modified by Chx or on Trusoft modified by It (p<0.001). However, Softone specimens modified by It and Trusoft modified by Chx exhibited higher roughness values than those observed at 24 h (p<0.001).

### *In vitro* leachability

The standard calibration curves with the corresponding linear regression equations for Chx (y=0.0188×+0.0258) and Ny (y=0.0157×+0.0141) related the absorbance values in UV-Vis to the drug concentration in the samples. The correlation coefficients obtained were 0.997 and 0.995 for Chx and Ny, respectively.

Based on the linear equations, the outcomes of daily release (µg/mL) of drugs from both materials were obtained. The greatest concentration released was observed on the first day of analysis. After 14 days, the tissue conditioner Softone released, on the average, 593.3 µg/mL of Chx and 323.6 µg/mL of Ny, whereas the resilient liner Trusoft released 521.2 µg/mL of Chx and 88.2 µg/mL of Ny. Softone leached a higher concentration of the antifungals than Trusoft (p=0.004), and chlorhexidine was released at a higher concentration than nystatin (p<0.001).

**Figure 6:**
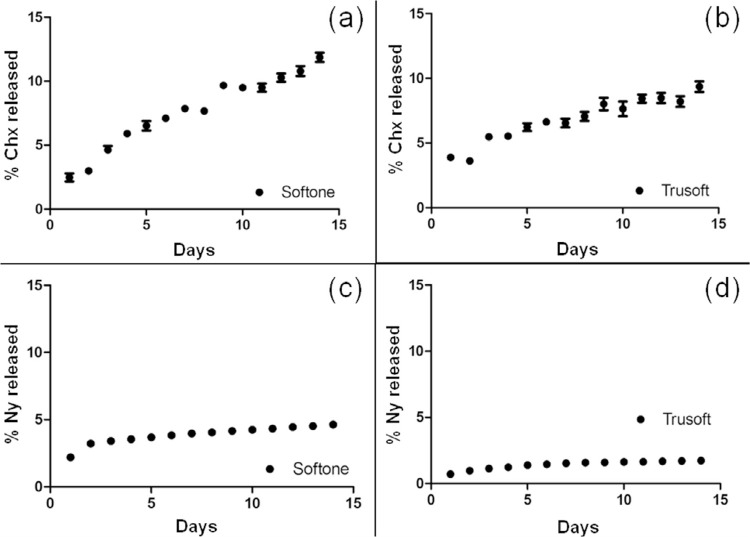
*In vitro* release profiles of Chx (a,b) and Ny (c,d) from Softone and Trusoft, respectively

The adjusted *in vitro* release profiles of Chx and Ny from Softone and Trusoft were obtained by plotting the percentages released with time ( Figure 6 ). Mathematical modeling data revealed that the best kinetics for both materials was explained by the biexponential model ( Table 1 ), with the materials showing a first stage of fast release (α) and a second one of slow or controlled release (β). The *n* values according to the Korsmeyer-Peppas test for Softone and Trusoft containing Chx were 0.6 and 0.36, respectively, and for the samples containing Ny, the *n* values were 0.22 and 0.29, respectively.

**Table 1:** Mathematical modeling by biexponential equation for both materials containing the drugs

Material	Drug	MSC	r	α(min^**-1**^)	β(min^**-1**^)
Softone	Chx	3.41	0.991	0.0053	0.3297
	Ny	5.45	0.998	17.729	0.0011
Trusoft	Chx	2.50	0.976	0.2389	0.0026
	Ny	5.25	0.999	0.0001	0.2967

### Scanning electron microscopy (SEM)

**Figure 7:**
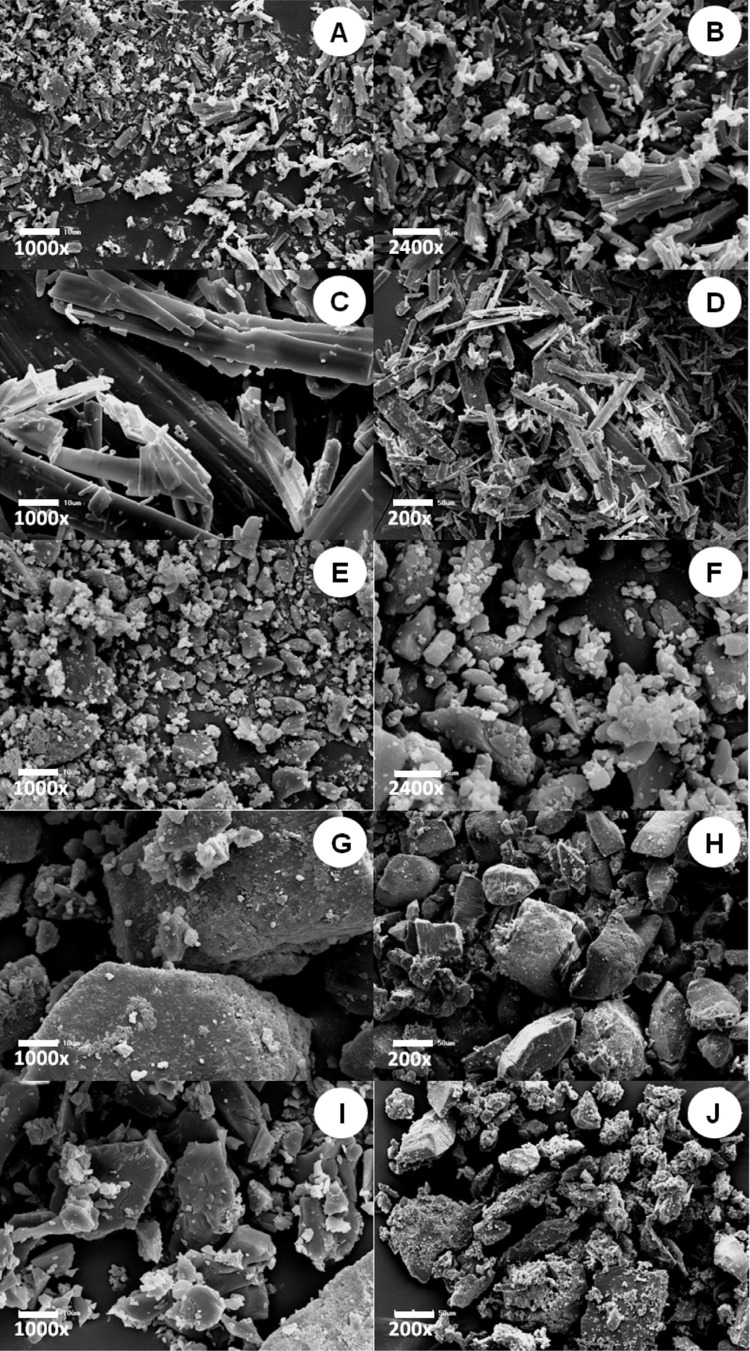
SEM images of drug particles. A and B – Ny; C and D – Mc; E and F – Ke; G and H – Chx; and I and J – It. Scale bars A, C, E, G, and I = 10 µm (Magnification 1000x); B and F = 5 µm (Magnification 2400x); and D, H, and J = 50 µm (Magnification 200x)

The SEM images of antifungal particles are presented in Figure 7 . The smallest particle sizes were observed for Ny and Ke. The particles of Ny presented irregular morphology and elongated shape with several sizes, smaller than 10 µm ( Figures 7A and 7B ). The particles of Ke had very reduced sizes (15 µm) and exhibited rounder shapes ( Figures 7E and 7F ). Mc exhibited particles with several sizes up to 200 µm with more elongated shape than Ny ( Figures 7C and 7D ). It exhibited higher particles with irregular characteristics, with appearance of spicules on its surface, with a maximum area of up to 150 µm^2^ ( Figures 7I and 7J ). Chx exhibited the highest particles, with a smoother aspect and a surface area of up to 150 µm^2^ ( Figures 7G and 7H ).

## Discussion

This study showed surface changes in both materials, especially in groups modified by the addition of antifungals and in samples immersed in water for longer periods. Therefore, the first hypothesis was accepted. One factor associated with the morphological surface change of soft materials is related to their degradation after immersion in aqueous solutions. Studies have reported that the release of alcohol and plasticizer in water may lead to an increase in the surface roughness of these materials after immersion times.^20^Moreover, the release of these components, which is accompanied by water absorption inside the soft material, leads to a loss of surface integrity.^21^ In addition to the inherent material degradation, the drug particles released to the medium can leave pores and empty spaces, yielding changes in their morphology.

Surface changes were observed with the addition of itraconazole, which may be attributed to the greater sizes and irregular shapes of its particles ( Figures 7I and 7J ), to the greater amount added to the materials^10^ and to the processing of this drug, which is commercially available as pellets. Additionally, though not measured, the water absorption affected its dimensional stability, since the volume increased in these modified specimens, which also changed their surface characteristics (Figures 3 and 4 – P to R). The surface changes of groups containing chlorhexidine diacetate and the increased roughness observed for the material Trusoft ( Figure 5 ) are consistent with previous studies. A previous study showed that a tissue conditioner modified by the incorporation of chlorhexidine acetate exhibited particles dispersed inside the polymeric matrix, which released antifungals for extended periods even at low doses.^22^ Despite the larger particle sizes ( Figures 7G and 7H ), a smaller quantity of chlorhexidine diacetate was necessary as MIC for the *C. albicans* biofilm;^10^ therefore, this drug should be considered in the treatment of denture stomatitis, because it shows some advantages over other antifungals. It presents broad-spectrum antimicrobial action, reaching bacteria and fungi present in the denture biofilm, besides exhibiting significant substantivity, which promotes effectiveness for longer study periods.^23^

Even though miconazole presented larger, yet finer particles ( Figures 7C and 7D ), it did not present apparent surface change compared to the control groups (Figures 3 and 4 – G to I). The modified material was more regular and had no pores. This may be due to the lower molecular weight of the miconazole particle when compared with other antifungals,^16^ which would allow greater diffusibility of this drug inside the polymeric matrix, leading to a higher solvation level.^24^ Both ketoconazole and nystatin exhibited smaller particle sizes ( Figures 7A and 7B, and 7E and 7F , respectively). Particles with smaller amount and size may diffuse more easily inside the polymeric matrix. Particularly, nystatin was added to the materials using the lowest MIC among the antifungals,^10^ because it presents the broader spectrum among available antifungals and is considered fungicidal.^6^

Materials with greater surface roughness may present a higher number of yeasts, since they may act as a microbial reservoir, increasing the resistance to shear forces during brushing.^25^ Therefore, ideally, soft materials for denture bases should present smooth surfaces to prevent the formation of biofilm and consequent inflammation of the oral mucosa and to facilitate hygiene, although roughness is not the only property related to microbial adhesion.^26^ In our study, the roughness values provided by the confocal microscope showed an increase at day seven, with a subsequent reduction at 14 days in distilled water to values lower than the initial value for the control groups and groups modified by the addition of nystatin, miconazole, and ketoconazole. The reduction in roughness with immersion in water may be associated with inherent characteristics of the material and to its composition. Immersion in water causes loss of soluble components, possibly leading to the formation of empty spaces and pores.^27^ Over time, these pores, which are responsible for the roughness, increase in size yielding craters; in turn, the edges of these craters are probably reduced and the specimens become smooth.^27^

Despite the surface changes observed after addition of drugs, micrographs of the control groups of soft materials analyzed (Figures 3A to 3C and 4A to 4C ) show that both materials presented potential for utilization as matrices for the addition and release of drugs to the intraoral environment, since they are permeable to fluids and present porosity, which are important factors related to the transportation of drug/water through the polymer. Additionally, studies have suggested that small changes in the physical and mechanical properties after the incorporation of antimicrobials, as observed on the surface analyses, might not interfere with the clinical performance of the materials^16^and would not contraindicate their utilization due to their advantage concerning the drug release, since temporary resilient linings and tissue conditioners are used for short periods.

The leachability study was only conducted with the modified materials by the addition of chlorhexidine diacetate and nystatin, which were highly effective in inhibiting the *C. albicans* biofilm at the lowest MICs.^10^ To be clinically effective, the drug added to polymeric systems should be released to the medium.^8^ Both evaluated antifungals were released to the aqueous environment during the study period. The chlorhexidine diacetate was released at a higher concentration than nystatin, and Softone presented greater capacity of release than Trusoft; thus the second hypothesis was accepted.

Nystatin, when added to the soft linings, presented inhibitory activity against *C. albicans* biofilm at only half the required quantity (0.032 g) for chlorhexidine diacetate (0.064 g) per gram of material powder.^10^ Since a lower concentration was added, a lower concentration could then be released. Other studies also observed this dose-dependent relationship, since the drug concentration released was directly proportional to the quantity added to the polymer.^9 , 28 , 29^

Despite exhibiting smaller particles than chlorhexidine diacetate ( Figures 7A and 7B, and 7G and 7H , respectively) that could facilitate its release due to the greater surface-area-to-volume ratio, nystatin presents greater molecular weight (926.11 versus 625.56 for chlorhexidine). Drugs with greater molecular weight require greater activation energy to penetrate and diffuse by the polymeric matrix, and that the rate of drug release may be increased by reducing its molecular weight.^30^ In addition to the lower concentration and greater molecular weight, nystatin also presents low solubility in hydrophilic solvents, yielding a slower release from the polymeric matrix.^29^ This study employed a solution of distilled water and 2% sodium lauryl sulfate to immerse the specimens containing nystatin. When surfactants were added to the medium for immersion of specimens modified by the addition of nystatin, we observed an easier release with greater concentration of surfactant. In this study, the surfactant sodium lauryl sulfate was added to the medium above the critical micelle concentration. Above this concentration, the surfactant forms micelles that may increase the solubility of substances that are poorly soluble in water.^29^ The surfactant also reduces the interfacial tension between polymer and dissolution medium, increasing the dispersibility of the matrix containing the drug, allowing its release. It also acts by promoting the entrance of fluid into the matrix, then dissolving and creating canals through which the drug may be released. Even with addition of surfactant, nystatin was released in low concentrations.

Although the manufacturer does not mention the concentration of plasticizers in the materials evaluated in study, according to the literature,^21^ it is expected that the tissue conditioner Softone present a greater quantity of plasticizer than that of the resilient liner Trusoft. Plasticizers reduce the glass transition temperature of the polymer, making the material soft.^24^ The plasticizer molecules, when released to the medium, leave pores in the material through which the drug may be released. Water absorption of the polymeric system, combined to its porous structure, allows controlled drug release, which may aid the treatment of persistent *Candida* infections.^28^ The greater release of both drugs from Softone matrix compared to Trusoft matrix would make allow the use Softone as vehicle for the incorporation and release of antifungals for the treatment of denture stomatitis.

The release kinetics of both drugs from the soft materials was biexponential, with high initial release followed by controlled release for 14 days. Other studies also revealed this pattern of release, greater in the first 24 h and controlled until the end of the study period.^8 , 28 , 30 , 31^ This release consists of two stages. The initial stage comprises an immediate effect (burst effect), which probably indicates the release of the drug on the material surface. The subsequent release may be the outcome of a complex process involving the formation of water droplets around the drug particles, in osmotically active sites, and the interaction of these droplets with the water absorption process^28 , 30^ and the polymer porosity.

According to the power law of the semi-empirical Korsmeyer-Peppas model,^19^ when the *n* values are lower than 0.43, drug release is controlled by diffusion (Fickian transport mechanism). When the *n* value is higher than 0.85, the release is controlled by swelling (or erosion) of polymer (case II transport or non-Fickian transport).^32^ Intermediate values 0.43< *n* <0.85 indicate mixed or abnormal behavior, involving both phenomena. The results showed that the mechanisms involved in nystatin release from both materials respected the Fick law, i.e., they involved only the diffusion process. This may be explained by the results from a prior study^11^, in which the addition of nystatin did not influence the water sorption and solubility of materials after 14 days of evaluation, indicating that this drug could be released without change in the fluid transport process through the matrices. Concerning chlorhexidine, different behaviors were observed between matrices: a Fickian transport mechanism was observed for the resilient liner Trusoft, and an abnormal mechanism was observed for the tissue conditioner Softone. Thus, the third hypothesis of this study was also accepted. These data may also be explained by the findings of a previous investigation,^11^ which showed that, after 14 days, only the material Softone modified by the incorporation of chlorhexidine at the same concentration as this study exhibited greater water sorption than the control groups. This indicates that there may have been swelling of the polymer, which, combined with the drug diffusion, enhanced the chlorhexidine release from Softone. Despite the greater drug release from this matrix, the subsequent volume change of the material might cause problems related to the dimension stability of the relined denture, requiring replacement of Softone after one week of utilization.

The results of this *in vitro* study should be carefully applied to clinical conditions. This study presents limitations, including simulation of the oral aqueous medium with salivary flow and renovation, and temperature and pH alterations. The addition of antifungal drugs in denture base temporary soft materials requires a final evaluation of their performance by *in vivo* studies.

## Conclusions

1. The tissue conditioner Softone exhibited more irregular surface morphology than the resilient liner Trusoft. Surface change with the increase in immersion time was observed in both materials, especially in those containing drugs, in which extremely porous and irregular surfaces were observed for groups containing chlorhexidine diacetate and itraconazole;
2. The specimens modified by the addition of drugs presented higher roughness values when compared with the control groups, that increased at 7 days, followed by a reduction to values lower than the initial ones at 14 days for the control group and those containing nystatin, miconazole, and ketoconazole;
3. Both materials presented biexponential release kinetics with fast initial release followed by a slower release. Softone released a higher concentration of drugs than Trusoft and chlorhexidine was released at a higher concentration than nystatin.
